# An 8-Channel Wavelength MMI Demultiplexer in Slot Waveguide Structures

**DOI:** 10.3390/ma9110881

**Published:** 2016-11-01

**Authors:** Bar Baruch Ben Zaken, Tal Zanzury, Dror Malka

**Affiliations:** Faculty of Engineering Holon, Institute of Technology (HIT), Holon 5810201, Israel; barbz096@gmail.com (B.B.B.Z.); talzanzury14@gmail.com (T.Z.)

**Keywords:** slot-waveguide, FV-BPM, MMI, DWDM

## Abstract

We propose a novel 8-channel wavelength multimode interference (MMI) demultiplexer in slot waveguide structures that operate at 1530 nm, 1535 nm, 1540 nm, 1545 nm, 1550 nm, 1555 nm, 1560 nm, and 1565 nm. Gallium nitride (GaN) surrounded by silicon (Si) was found to be a suitable material for the slot-waveguide structures. The proposed device was designed by seven 1 × 2 MMI couplers, fourteen S-bands, and one input taper. Numerical investigations were carried out on the geometrical parameters using a full vectorial-beam propagation method (FV-BPM). Simulation results show that the proposed device can transmit 8-channel that works in the whole C-band (1530–1565 nm) with low crosstalk (−19.97–−13.77 dB) and bandwidth (1.8–3.6 nm). Thus, the device can be very useful in optical networking systems that work on dense wavelength division multiplexing (DWDM) technology.

## 1. Introduction

Dense wavelength division multiplexing (DWDM) is an optical multiplexing technology used to increase the bandwidth over existing fiber networks [1]. DWDM works by combining and transmitting multiple signals simultaneously at different wavelengths on the same fiber [2].

An optical demultiplexer is an important component in optical communication networks that work on DWDM technology. Demultiplexers can be implemented using several techniques, such as Mach-Zehnder interferometers [3], multimode interference (MMI) couplers [4,5,6], and Y-branch devices [7].

A slot-waveguide is a unique structure that enables light to be strongly confined and guided inside a narrow nanometer-scale region of low index material that is surrounded by two layers with high index material [8].

Using this unique structure leads to a variety of advantages such as a small beat length of the guided light and a strong confinement in the slot region that results in extremely low losses. Another benefit is that CMOS compatible materials and technology can be used in slot-waveguide fabrication [9].

A major improvement in the fabrication of semiconductor circuits is the introduction of silicon on insulator (SOI) technology. This technology is characterized by low power consumption, improved heat dissipation, and low-voltage activity. As a result, the performance of semiconductor circuits has improved significantly [10,11].

The principle work of the MMI coupler is that an input field is duplicated in single or multiple images at periodic intervals along the light propagation in the MMI waveguide coupler. This effect is called self-imaging [12,13].

MMI demultiplexers based on slot waveguide structures have been demonstrated to separate two channels with a spacing of 250 nm [14] and four channels with a spacing of 50 nm [15].

The wavelengths, ranging from 1530 to 1565 nm, are the most useful range in optical telecommunication, and it is called the C-band. The main benefit of the C-band is the use of optical amplifiers that allows for the transmission of data over long distances [16].

Gallium nitride (GaN) has some important electrical characteristics including a wide spectral range and a resistance to temperature [17,18]. GaN devices can be grown epitaxially on substrates or can be grown directly on silicon (Si) substrates [19].

Researchers assessed the performance of GaN-based slot-waveguide device and found it suitable for transmitting visible light with 0.1–0.4 (dB/cm) transmission loss [20].

The preliminary fabrication of 1 × 4 power splitter based on MMI in Si-GaN slot waveguide structures has been demonstrated [21], and researchers have recently made an initial demonstration of GaN CMOS field-effect-transistor technology to fabricate a functional inverter integrated circuit [22].

An MMI demultiplexer device based on conventional Si waveguides demonstrated an ability to separate eight channels with a spacing of 5 nm in the C-band [23]. However, the simulation results show low transmission values of 42.6%–57% and a large coupling length size (18 mm) along the z-axis.

Choosing a lower-index material as the slot material lead to a stronger confinement inside the slot area. However, an MMI demultiplexer device with close spacing channels is very sensitive to the variation in the effective refractive index, which can influence the performance and especially the MMI coupler size.

In order to overcome this problem, we chose GaN as a slot material. GaN has a high-index value compared to other materials (alumina or silica (SiO_2_)) and has a low-index value compared with Si material.

Thus, the MMI demultiplexer device-based Si-GaN slot waveguide is not very sensitive to the variation in the optical signals in the C-band [20] that enable an ability to separate wavelengths in the C-band inside the MMI coupler with improved performance. 

In this paper, we present a 1 × 8 wavelength MMI demultiplexer in a slot Si-GaN waveguide structure that divides eight channels in the C-band range with a spacing of 5 nm between channels. The operating wavelengths are: 1530 nm, 1535 nm, 1540 nm, 1545 nm, 1550 nm, 1555 nm, 1560 nm, and 1565 nm. Thus, this device can be very useful for transmitting a wide range of information in DWDM systems.

The device is based on a cascade of seven 1 × 2 MMI couplers, fourteen S-bands, and one input taper. Numerical optimizations were carried out on the MMI coupler parameters and the slot-waveguide structure in order to obtain strong field confinements inside the slot region, a self-image effect, and to find the optimal values of the MMI couplers. The simulations were done using the full vectorial-beam propagation method (FV-BPM) combined with Matlab software.

## 2. The 1 × 8 MMI Demultiplexer Structure and Theoretical Aspect

**Figure 1:**
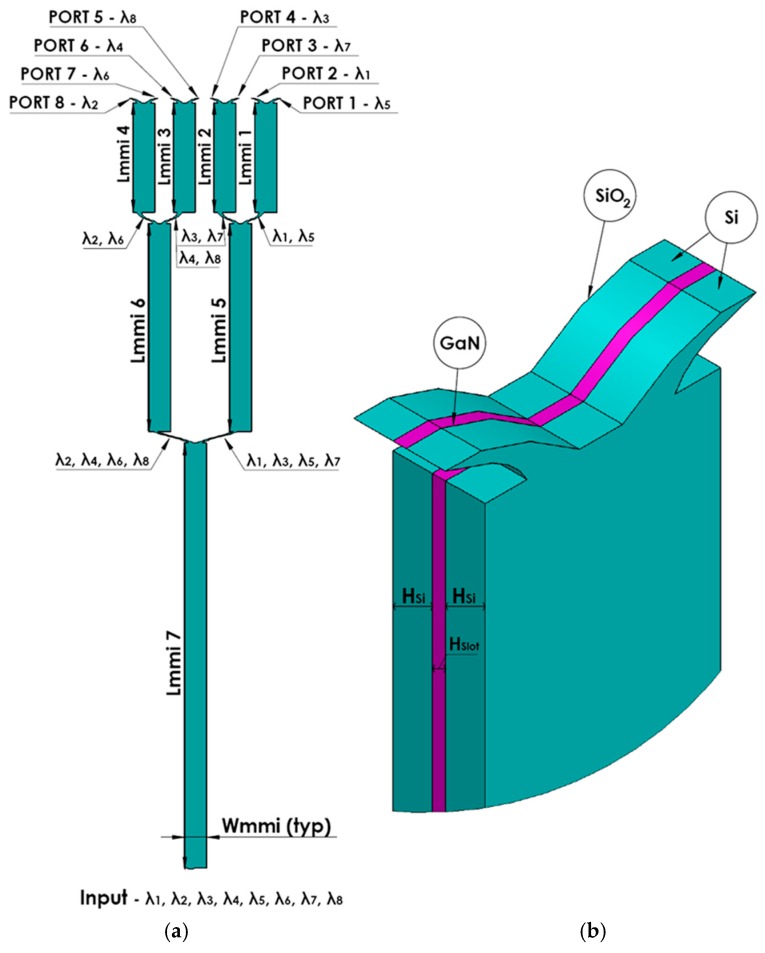
Schematic sketch of the 1 × 8 wavelength MMI demultiplexer: (**a**) x–z plane; (**b**) MMI coupler 3D view with zoom.

Figure 1a,b shows a schematic sketch of the 1 × 8 wavelength MMI demultiplexer x–z cross sectional view at y = 0 and 3D view of the MMI coupler. In this figure, the green areas denote pure silicon (Si), purple areas denote GaN, and the white areas denote silica (SiO_2_). The Si layer height is H_Si_, and the GaN layer height is H_slot_ as shown in Figure 1b. It can be seen in Figure 1a that the device is based on seven 1 × 2 MMI couplers, fourteen S-bands, and one input taper. Table 1 shows the refractive index values of Si, GaN, and SiO_2_ at the operated wavelengths.

The width of the input taper varies from 0.4 µm to 0.6 µm with a length of 60 µm. The width of the output S-band is 0.4 µm and varies from 0.35 µm to 0.4 µm for the left and right outputs in the MMI coupler. The gap distance between the two S-bands at the output MMI coupler is 0.73 µm.

The MMI coupler is based on the self-imaging effect of multimode interference [24,25]. The beat length L_π_ is given by [24]

(1) $$L_{\pi ,m}\approx \frac{4n_{\mathrm{eff}}\left(\lambda_{m}\right){W_{e,m}}^{2}}{3\lambda_{m}};m=1,2,3,...,8.$$

λ_m_ are the operating wavelengths ($\lambda_{m}=\sum_{m=1}^{8}1525+5m$ (nm)). The n_eff_ (λ_m_) is the effective refractive index of the core (GaN and Si) and is solved by the FV-BPM mode solver. The W_e,m_ is the effective width of the MMI couplers; for the transverse magnetic (TM) mode, the W_e,m_ is approximated by [24]

(2) $$W_{e,m}=W_{MMI}+\frac{\lambda_{m}}{\pi }{\left(\frac{n_{SiO2}(\lambda_{m})}{n_{eff}(\lambda_{m})}\right)}^{2}\frac{1}{{\left(n_{eff}^{2}(\lambda_{m})-n_{SiO2}^{2}(\lambda_{m})\right)}^{0.5}},$$

where W_MMI_ is the width of the MMI coupler as shown in Figure 1b. Its size was optimized in order to minimize the size of the beat length inside the MMI coupler.

In order to obtain the directed or the mirrored image of the entered field at the output coupler, the MMI coupler length needs to be equal to a natural number duplicated with the beat length (L_mmi_ = pLπ).

The conditions for dividing two different wavelengths in MMI coupler are given by

(3) $$\begin{array}{l} L_{mmi,1}=p_{1}L_{\pi }^{\lambda_{1}}=(p_{1}+q_{1})L_{\pi }^{\lambda_{5}}\\ L_{mmi,2}=p_{2}L_{\pi }^{\lambda_{3}}=(p_{2}+q_{2})L_{\pi }^{\lambda_{7}}\\ L_{mmi,3}=p_{3}L_{\pi }^{\lambda_{4}}=(p_{3}+q_{3})L_{\pi }^{\lambda_{8}}\\ L_{mmi,4}=p_{4}L_{\pi }^{\lambda_{2}}=(p_{4}+q_{4})L_{\pi }^{\lambda_{6}}, \end{array}$$

where p is a natural number, and q is an odd number. The conditions for dividing four different wavelengths in MMI coupler are given by

(4) $$\begin{array}{l} L_{mmi,5}=p_{5}L_{\pi }^{\lambda_{1}}=\left(p_{5}+q_{5}\right)L_{\pi }^{\lambda_{3}}=\left(p_{5}+q_{5}+1\right)L_{\pi }^{\lambda_{5}}=\left(p_{5}+q_{5}+2\right)L_{\pi }^{\lambda_{7}}\\ L_{mmi,6}=p_{6}L_{\pi }^{\lambda_{2}}=\left(p_{6}+q_{6}\right)L_{\pi }^{\lambda_{4}}=\left(p_{6}+q_{6}+1\right)L_{\pi }^{\lambda_{6}}=\left(p_{6}+q_{6}+2\right)L_{\pi }^{\lambda_{8}}. \end{array}$$

The conditions for dividing eight different wavelengths in MMI coupler are given by

(5) $$\begin{array}{l} L_{mmi,7}=p_{7}L_{\pi }^{\lambda_{1}}=(p_{7}+q_{7})L_{\pi }^{\lambda_{2}}=\left(p_{7}+q_{7}+1\right)L_{\pi }^{\lambda_{3}}=\left(p_{7}+q_{7}+2\right)L_{\pi }^{\lambda_{4}}=\left(p_{7}+q_{7}+3\right)L_{\pi }^{\lambda_{5}}\\ =\left(p_{7}+q_{7}+4\right)L_{\pi }^{\lambda_{6}}=\left(p_{7}+q_{7}+5\right)L_{\pi }^{\lambda_{7}}=\left(p_{7}+q_{7}+6\right)L_{\pi }^{\lambda_{8}}. \end{array}$$

In order to obtain a compact device, the location of the input taper was shifted $\pm \frac{1}{6}\mathrm{We}$ from the center of W_mmi_. This shift can lead to a cancellation of the third mode inside the MMI coupler. In addition, many optimizations were carried to find the optimal values of the seven MMI coupler lengths that satisfied the conditions in Equations (3)–(5).

The crosstalk is given by

(6) $$C.T_{n}=\frac{1}{7}\sum_{m=1}^{8}10log(\frac{P_{m}}{P_{n}}),$$

where P_n_ is the power transmission for the suitable port, and P_m_ is the interference power transmission from the other port. The insertion losses are given by

(7) $$Losses(dB)=-10Log_{10}\left(\frac{p_{out}}{P_{in}}\right),$$

where P_out_ is the power at the output port, and P_in_ is the power in the input taper.

## 3. Results

**Figure 2:**
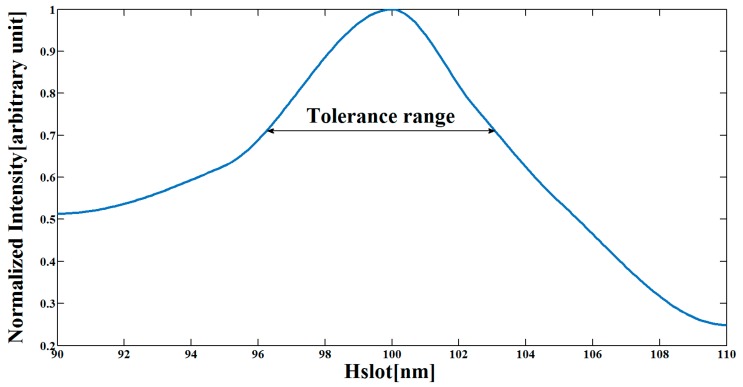
Normalized intensity as function of H_Slot_.

The simulations were done using a FV-BPM-based RSoft Photonics CAD Suite software. The optimal values of the slot-waveguide structure were calculated by FV-BPM simulations combined with Matlab software. The optimal values are H_Si_ = 300 nm, H_Slot_ = 100 nm, and W_mmi_ = 1.8 µm. Figure 2 shows the normalized intensity in the slot area as function of H_Slot_. The optimal tolerance values of H_Slot_ were set between 70%–100% of the normalized intensity (black line in Figure 2).

From Figure 2, it can be noticed that the tolerance values of H_Slot_ are around 6–7 nm.

**Figure 3:**
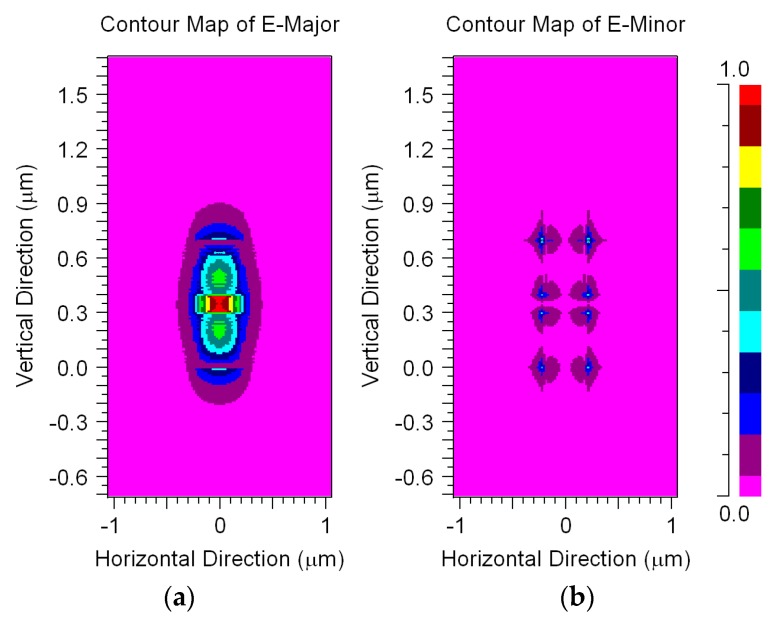
Field patterns of the quasi-TM fundamental mode for the device: (**a**) Ey; (**b**) Ex.

Figure 3a,b show the field patterns of the quasi-TM fundamental mode at 1.55 µm. It can be seen in Figure 3a that there are no confinement losses due to the strong confinement of the electric field (Ey) inside the slot area (red color). A similar mode profile field was obtained for the other operated wavelengths.

The values of neff (λm) were found by solving the field mode profile. By solving Equations (1) and (2), the values of the beat length for the operated wavelengths (see Table 2) can be found.

It can be seen in Table 2 that the variation of the beat length value is only 170 nm in the C-band range.

**Figure 4:**
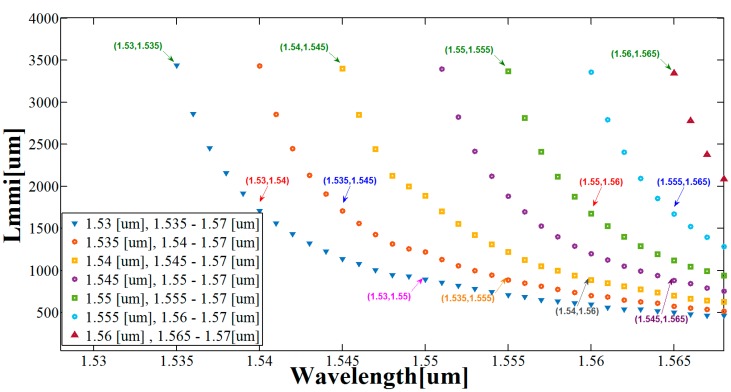
Lengths of the MMI couplers as a function of the wavelength pairs around the C-band range.

Figure 4 shows the lengths of the seven MMI couplers that satisfy the conditions in Equations (3)–(5). The wavelength pairs (around the C-band) values are 1.53 µm, 1.535–1.57 µm (blue triangles); 1.535 µm, 1.54–1.57 µm (red circles); 1.54 µm, 1.545–1.57 µm (yellow rectangles); 1.545 µm, 1.55–1.57 µm (purple circles); 1.55 µm, 1.555–1.57 µm (green rectangles); 1.555 µm, 1.56–1.57 µm (light blue circles); 1.56 µm, 1.565–1.57 µm (brown triangles).

Based on numerical optimizations combined with Equations (3)–(5), the appropriate values of the seven lengths of the MMI couplers can be found. Their values are Lmmi,1 = 892.36 µm, Lmmi,2 = 887.25 µm, Lmmi,3 = 885 µm, Lmmi,4 = 889.947 µm, Lmmi,5 = 1.72 mm, Lmmi,6 = 1.688 mm, and Lmmi,7 = 3.451 mm. We chose these wavelengths because they have the best approximation for Lmmi,7, which is suitable for four wavelength pairs that belong to the C-band range. Lmmi,7 (green arrows in Figure 4) is suitable for λ1, λ2 (blue triangle), λ3, λ4 (yellow rectangle), λ5, λ6 (green rectangle), and λ7, λ8 (brown triangle); Lmmi,6 (blue arrows in Figure 4) is suitable for λ2, λ4 (red circle), and λ6, λ8 (light blue circle); Lmmi,5 (red arrows in Figure 4) is suitable for λ1, λ3 (blue triangle) and λ5, λ7 (green rectangle); Lmmi,4 (orange arrow in Figure 4) is suitable for λ2, λ6 (red circle); Lmmi,3 (purple arrow in Figure 4) is suitable for λ4, λ8 (purple circle); Lmmi,2 (gray arrow in Figure 4) is suitable for λ3, λ7 (yellow rectangle); Lmmi,1 (pink arrow in Figure 4) is suitable for λ1, λ5 (blue triangle).

**Figure 5:**
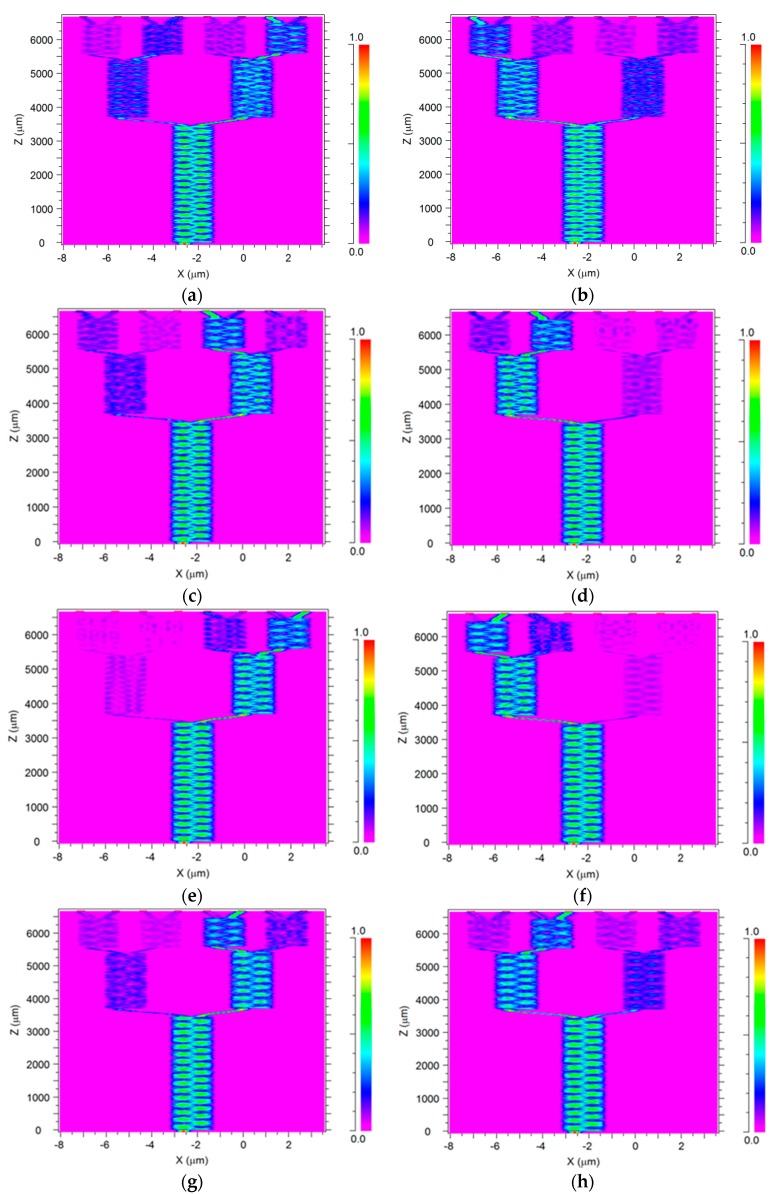
Intensity profile of the 1 × 8 MMI wavelength demultiplexer: (**a**) λ1 = 1530 nm (Port 2); (**b**) λ2 = 1535 nm (Port 8); (**c**) λ3 = 1540 nm (Port 4); (**d**) λ4 = 1545 nm (Port 6); (**e**) λ5 = 1550 nm (Port 1); (**f**) λ6 = 1555 nm (Port 7); (**g**) λ7 = 1560 nm (Port 3); (**h**) λ8 = 1565 nm (Port 5).

Figure 5a–h show the intensity profile of the optical signals at the x–z plane. The first MMI coupler divides eight wavelengths (λ2, λ4, λ6, λ8 and λ1, λ3, λ5, λ7) at z = 3.5 mm; the second MMI coupler divides four wavelengths (λ2, λ4) and (λ4, λ8) as shown in Figure 5b,d,f,h at z = 5.5 mm; the third MMI coupler divides four wavelengths (λ1, λ3 and λ5, λ7) as shown in Figure 5a,c,e,g at z = 5.4 mm; the fourth MMI coupler divides two wavelengths (λ2 and λ6) as shown in Figure 5b,f at z = 6.6 mm; the fifth MMI coupler divides two wavelengths (λ4 and λ8) as shown in Figure 5d,h at z = 6.6 mm; the sixth MMI coupler divides two wavelengths (λ3 and λ7) as shown in Figure 5c,g at z = 6.6 mm; the seventh MMI coupler divides two wavelengths (λ1 and λ5) as shown in Figure 5a,e at z = 6.6 mm.

It can be seen in Figure 5a–h that the coupling length along the z-axis is 6.6 mm. This value indicates that this device has a compact size compared with the MMI demultiplexer device based on conventional Si waveguides [23].

**Figure 6:**
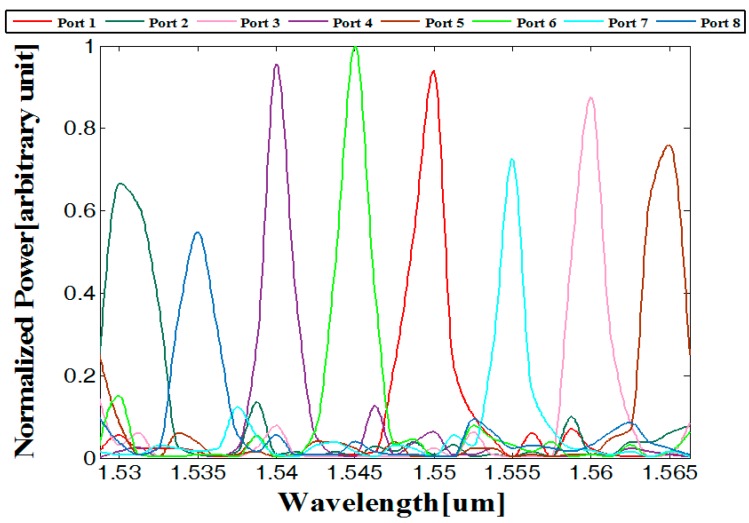
Normalized power as function of the operated wavelengths.

FV-BPM simulations combined with Matlab code was performed to determine the 1 × 8 wavelength MMI demultiplexer properties. Figure 6 shows the spectral transmission results for the wavelengths around the C-band range (1530–1565 nm).

By solving Equations (6) and (7), combined with the results of Figure 6, the values of the crosstalk, full width maximum (fwhm), and insertion losses can be found. Table 3 shows the values of the crosstalk, bandwidth (fwhm), and loss for each port.

## 4. Conclusions

To summarize, in this paper, we have shown that a 1 × 8 wavelength MMI demultiplexer can be implemented in slot Si-GaN waveguide structures.

Simulation results show that eight wavelengths—1530, 1535, 1540, 1545, 1550, 1555, 1560, and 1565 mm—that belong to the C-band range can be divided after a propagation length of 6.6 mm with insertion losses in the range of 0.9–2.12 dB.

We managed to shorten the coupling distance along the z-axis from 18 mm [23] to 6.6 mm.

The device has low crosstalk (−19.97–−13.77 dB), with a bandwidth range of 1.8–3.6 nm. Therefore, this device can be very useful in optical networking systems that work on DWDM technology.

Although only the demultiplexer configuration is considered in this manuscript, the demultiplexer can also operate as a multiplexer in a reversed direction of the guided light.

Due to the use of the slot Si-GaN waveguide structure, the device has great potential for integration with CMOS technology for the design of a photonic-chip.

## Figures and Tables

**Table 1:** The slot waveguide materials reflective index values.

λ_m_ (nm)	1530	1535	1540	1545	1550	1555	1560	1565
n_Si_	3.4794	3.4790	3.4786	3.4781	3.4777	3.4773	3.4769	3.4765
n_SiO_2__	1.4443	1.4442	1.4441	1.4441	1.444	1.444	1.4439	1.4438
n_GaN_	2.3173	2.3172	2.3171	2.3170	2.3169	2.3168	2.3167	2.3165

**Table 2:** The beat length values.

**λ_m_ (nm)**	1530	1535	1540	1545	1550	1555	1560	1565
**L_π_ (µm)**	9.295	9.27	9.245	9.22	9.2	9.175	9.15	9.125

**Table 3:** Values of the crosstalk, fwhm, and losses for the operated wavelengths.

λ_m_ (nm)	1530	1535	1540	1545	1550	1555	1560	1565
Port number	2	8	4	6	1	7	3	5
Crosstalk (dB)	−19.97	−19.53	−18.89	−18.04	−18.86	−19.41	−18.7	−13.77
FWHM (nm)	2.35	3.6	2.35	1.95	2.8	2.2	1.8	2.9
Losses (dB)	1.78	2.12	1.2	0.9	1.32	1.71	1.45	1.67

## References

[1] Bogdan H. (2002). DWDM Fundamentals, Components, and Applications. J. Opt. Netw..

[2] Gong J.M., Zuo X., Zhao Y. (2015). The steady SRS analysis theory of DWDM transmission system in single-mode silica fiber. Opt. Commun..

[3] Ari T., Petteri P., Seppo H., Markku T. (1991). A guided-wave Mach-Zehnder interferometer structure for wavelength multiplexing. IEEE Photon. Technol. Lett..

[4] Lin K.C., Lee W.Y. (1996). Guided-wave 1.30/1.55 µm wavelength division multiplexer based on multimode interference. Electron. Lett..

[5] Li B., Li G., Liu E., Jiang Z., Qin J., Wang X. (1999). Low-loss 1 × 2 multimode interference wavelength demultiplexer in silicon-germanium alloy. IEEE Photon. Technol. Lett..

[6] Tsao S.L., Guon H.C., Tsai C.W. (2004). A novel 1 × 2 single-mode 1300/1550 nm wavelength division multiplexer with output facet-tilted MMI waveguide. Opt. Commun..

[7] Goto N., Yip G.L. (2007). Y-branch wavelength multi-demultiplexer for λ = 1.30 µm and 1.55 µm. Electron. Lett..

[8] Almeida V.R., Xu Q., Barrios C.A., Lipson M. (2004). Guiding and confining light in void nanostructure. Opt. Lett..

[9] Zhu S., Liow T.Y., Lo G.Q., Kwong D.L. (2011). Silicon-based horizontal nanoplasmonic slot waveguide for on-chip integration. Opt. Express.

[10] Soref R. (2006). The past, present, and future of silicon photonics. IEEE J. Sel. Top. Quantum Electron..

[11] Jalai B., Fathpour S. (2006). Silicon photonics. J. Lightwave Technol..

[12] Olof B. (1973). Image formation using self-imaging techniques. J. Opt. Soc. Am..

[13] Fujisawa T., Koshiba M. (2006). Theoretical Investigion of ultrasmall polarization-insensitive multimode interference waveguide based on sandwiched structures. IEEE Photon. Technol. Lett..

[14] Xiao J., Liu X., Sun X. (2007). Design of an ultracompact MMI wavelength demultiplexer in slot waveguide structures. Opt. Express.

[15] Malka D., Sintov Y., Zalevsky Z. (2015). Design of a 1 × 4 silicon-alumina wavelength demultiplexer based on multimode interference in slot waveguide structures. J. Opt..

[16] Dike J.N., Ogbe D.A. Optimizing the Efficiency of Fiber-Optics Technology in Telecommunications System. Proceedings of the IEEE International Conference on Emerging & Sustainable Technologies for Power & ICT in Developing Society (NIGERCON).

[17] Retno W.P., Irma S., Nji Raden P., Elhadj D. (2014). Design of GaN-Based Low-Loss Y-Branch Power Splitter. Makara J. Technol..

[18] Komatsu M.A., Saitoh K., Koshiba M. (2013). Design of highly-nonlinear horizontal slot waveguide with low and flat dispersion. Opt. Commun. J. Sci. Direct.

[19] Carnevale S.D., Kent T.F., Phillips P.J., Mills M.J., Rajan S., Myers R.C. (2012). Polarization-induced pn diodes in wide-band-gap nanowires with ultraviolet electroluminescence. Nano Lett..

[20] Xiao X., Li X., Fen X., Cui K., Liu F., Huang Y. (2015). Designing gallium nitride slot waveguide operating within visible band. Opt. Quant Electron..

[21] Malka D., Danan Y., Ramon Y., Zalevsky Z. (2016). A Photonic 1 × 4 Power Splitter Based on Multimode Interference in Silicon-Gallium-Nitride Slot Waveguide Structures. Materials.

[22] Chu R., Cao Y., Chen M., Li R., Zehnder D. (2016). An Experimental Demonstration of GaN CMOS Technology. IEEE Electron Device Lett..

[23] Rostami A., Bahrami A., Nazari F., Alupour Banaei H. Eight-channel wavelength division demultiplexer using multimode interference. Proceedings of the Communications and Photonics Conference and Exhibition (ACP).

[24] Almedia V.R., Barrios C.A., Panepucci R.R., Lipson M. (2004). All-optical control of light on a silicon chip. Nature.

[25] Soldano L.B., Pennings E.C.M. (1995). Optical multimode interference devices based on self-imaging: Principles and applications. J. Lightwave Technol..

